# Repositioning the Chemical Information Science Gateway

**DOI:** 10.12688/f1000research.19764.1

**Published:** 2019-06-27

**Authors:** Jürgen Bajorath

**Affiliations:** 1 University of Bonn, Bonn, Germany

**Keywords:** Chemical information science, gateway design

## Abstract

The Chemical Information Science Gateway (CISG) of F1000Research was originally conceptualized as a forum for high-quality publications in chemical information science (CIS) including chemoinformatics. Adding a publication venue with open access and open peer review to the CIS field was a prime motivation for the introduction of CISG, aiming to support open science in this area. Herein, the CISG concept is revisited and the development of the gateway over the past four years is reviewed. In addition, opportunities are discussed to better position CISG within the publication spectrum of F1000Research and further increase its visibility and attractiveness for scientific contributions.

## Scientific background

Chemical information science (CIS) includes all computational approaches to represent and analyze chemical structures and chemical data or retrieve chemical information from any source ^1^. The more narrowly defined chem(o)informatics discipline focuses on the organization and systematic analysis of small molecules, associated experimental data, and structure-property relationships as well as on the design and prediction of new chemical entities with desired properties ^2– 4^. In addition, chemoinformatics addresses emerging ‘big data’ trends in chemistry ^5, 6^ and covers artificial intelligence methods, which experience a new wave of excitement in many scientific fields including chemistry ^7, 8^. Notably, machine learning methods have a long history in chemoinformatics and are a mainstay for compound property prediction ^4^. Given its scope and methodological repertoire, chemoinformatics can be rationalized as a part of the CIS spectrum ^1, 9^. 

## Evolution of the chemical information science gateway

In 2015, the CIS Gateway (CISG) was originally launched as a CIS publication ‘Channel’ by F1000Research ^9^ to provide a new open access and open peer review ^10^ platform for broadly defined CIS publications. It was considered a much-needed extension of the CIS journal landscape with emphasis on open science and transparent peer review ^9^. In 2017, the CIS Channel became a Gateway, its scope was further refined, and its advisory board extended ^11^, leading to CISG in its current form. Without doubt, CISG filled a void within the journal landscape. In addition to fostering open science and post-publication communication, the CISG initiative strongly emphasized contributions of high scientific quality. Therefore, a two-stage review was established. Submissions to CISG were pre-reviewed by its advisory board to determine if the work had scientific quality and potential to advance the field. If so, the manuscript was published in CISG and subjected to open peer review using referees agreed upon by the authors. If not, authors still had the opportunity to publish their work in F1000Research outside CISG. Furthermore, the submission of off-the-beaten-path contributions to CISG was strongly encouraged ^9, 11^, for example, studies showing why a computational method was not capable of solving a particular problem. From the beginning, the CISG concept was enthusiastically received by investigators in the field and a number of leading experts joined CISG’s advisory board. Hence, CISG was off to a good start, with strong support from F1000Research.

## The first years in review

In the past four years, CISG has not attracted the attention in the greater CIS community we initially had hoped for. So far, only 16 papers have been published in CISG (including only one in 2018). The number of papers was never regarded as a primary success criterion for CISG, but steady growth was desirable, as an indicator of increasing awareness and acceptance in the field. While it is generally difficult to establish new journals or publication platforms, more positive resonance was anticipated. Some CISG advisors have explored reasons for the apparent reluctance to publish in CISG and a few likely causes emerged. First and foremost, the absence of an impact factor (IF) –consistent with the publication philosophy of F1000Research– presents a problem for scientists whose advancement largely depends on IF criteria. This affects entire academic communities (such as in China). While views concerning IFs greatly differ in the field, IF-dependent publication restrictions cannot be disregarded and inevitably confine the number of potential authors. In addition, required article processing charges for open access (which are moderate at F1000Research) appear to play a role for many in the CIS field, more so than one might expect. Moreover, perhaps surprisingly, a number of investigators do not feel comfortable with open peer review, whereas others strongly support it. However, there still is a tendency to shy away from exposing scientific work to public evaluations, although open review and scientific discourse need to follow defined criteria and standards ^10, 12^. On the other hand, open review might occasionally also come across as too timid. Taken together, these points indicate that more efforts –and more publications– will be required to establish an open review culture in the CIS field that rivals anonymous peer review. 

The considerations above concerning IF, article processing charges, and open review are general in nature and not specific to CISG. However, in combination, these issues have made it difficult to establish CISG on the CIS publication landscape, although it is rather sparsely populated with specialized journals (with low to moderate IFs). Moreover, there are at least two CISG-specific issues that might have hindered its evolution. First, the two-stage review process is frequently viewed controversially and might be considered as overly strict. Second, CISG has occasionally been regarded as a quasi “private” or “elite” publication venue, which was, of course, far from being the intention. The quest for high scientific quality was never considered the privilege of just a few. Regardless, these issues have most likely also contributed to the slow development of CISG over the past four years, while a flagship journal in the CIS field is currently missing.

On a positive note, since launching CISG, open science in the traditionally conservative chemical sciences has further progressed. For example, new open access journals have been introduced by major publishers and there is a clear trend to make chemical data and software tools publicly available on open access platforms (in part due to more stringent data deposition requirements for publications). In addition, many academic-industry collaborations have been initiated in drug discovery, material science, and nanoscience, and CIS (focusing on artificial intelligence), which also help to support open science initiatives. Furthermore, with the recent announcement of Plan S ^13^, major funding agencies including the EU commission will now require open access dissemination of research results including the chemical sciences. F1000Research in particular has seen a growth in submissions since 2015 as open access publishing becomes more ‘mainstream’.

## Entering the next phase

Despite the complications discussed above, reasons for further supporting and promoting CISG remain unchanged and are as attractive as at the time of CISG’s inception: open science, open access, fast publication, transparent reviews, and ample opportunities for unconventional contributions. While we will not be able to address IF-related publication constraints, going forward, some adjustments will be made to further increase the visibility of CISG and its attractiveness as a publication venue.

CISG will continue to build upon its high-calibre advisory board and emphasize high-quality science. However, we will soften the two-stage review process and pre-review criteria. Only if there is a consensus view among advisors that a submission is scientifically not suitable for CISG, it will be forwarded to F1000Research for further consideration. Otherwise, the manuscript will be published immediately in CISG and subjected to open peer review, with the help of advisors. Several early career researchers will be added to the advisory board to further increase its range of scientific interests, motivations, and experiences. 

Importantly, other Gateways and Collections have emerged on the F1000Research platform, which are related to CISG from a methodological viewpoint, such as the *Cytoscape Apps* ^14^ and *RPackage* ^15^ Gateways or the *Bioinformatics Education and Training* ^16^ and the *Python* ^17^ Collections. For example, Python has become the preferred programming language for CIS. These related Gateways and Collections including CISG may be more closely linked going forward and positioned in context, offering opportunities for cross-over calls for papers or other joint initiatives. For example, given the increasing emphasis put on data science and artificial intelligence in chemistry, biology, and drug discovery, themed calls for contributions focusing on data science and artificial intelligence in different fields might be considered. F1000Research’s staff will launch and coordinate such efforts, which will provide many opportunities for submission of Method Articles, Software Tool Articles, Data Notes, or other types of papers, allowing researchers credit for the range of research outputs they are producing. Given the close connection between the Python Collection and programing in CIS, there should also be significant potential for growth in this area, especially for publication of Software Tool Articles. F1000Research have worked closely with software communities to help support computational paper submissions more broadly by allowing authors to submit in LaTeX ^18^, integrate interactive figures into articles via Plotly ^19^ and be able to run code re-analysis on the article page ^20^. In addition, Data Notes will become increasingly important for CISG, given its close link to data science, similar to developments in computational biology.

We are looking forward to seeing CISG entering its next phase, hopefully with more resonance from the CIS field. It is noted that CISG continues to provide the only opportunity for open review in CIS and we hope that more investigators will see the benefits of the open post-publication review culture. From the CISG access statistics, it is clearly evident that articles published there are seen and read. From our own contributions to CISG, I know that they are widely cited as well, which is more important than an IF label. Hence, there are good reasons to give it a try and submit to CISG, with the added bonus of efficient handling of papers by F1000Research and its streamlined editorial procedures. 

## Data availability

No data are associated with this study.
